# Recession of Environmental Barrier Coatings under High-Temperature Water Vapour Conditions: A Theoretical Model

**DOI:** 10.3390/ma13204494

**Published:** 2020-10-10

**Authors:** Peng Jiang, Cheng Ye

**Affiliations:** State Key Laboratory for Strength and Vibration of Mechanical Structures, Xi’an Jiaotong University, Xi’an 710049, China; yecwizards@stu.xjtu.edu.cn

**Keywords:** environmental barrier coating, high-temperature water-vapour recession, ytterbium disilicates

## Abstract

Rare-earth disilicates are the major material used on the top layer of environmental barrier coating (EBC) systems. Although rare-earth disilicates are highly resistant to water vapour, corrosion due to water vapour at high temperature is still one of the main reasons of failure of EBC systems. In this study, a corrosion model of ytterbium disilicates in water vapour at high temperature was derived, based on the gas diffusion theory. Using this theoretical model, we studied the evolution rule of the corroded area on the top layer of the EBC under gas flow at high temperature. The influence of the various parameters of the external gas on the corrosion process and the corrosion kinetics curve were also discussed. The theoretical model shows that the increase in gas temperature, gas flow velocity, water partial pressure, and total gas pressure accelerate coating corrosion. Among these factors, the influence of total gas pressure on the corrosion process is relatively weak, and the effect of the continuous increase of the gas velocity on the corrosion process is limited. The shape of the corrosion kinetics curve is either a straight or parabolic, and it was determined by a combination of external gas parameters.

## 1. Introduction

Continuous silicon carbide fibre-reinforced silicon carbide ceramic matrix composite (SiC/SiC CMC) has been widely used in high-temperature environments owing to its high-temperature resistance, excellent mechanical properties, and low density [1,2,3]. Nowadays, SiC/SiC CMC is gradually replacing the traditional nickel-based superalloy as the main structure and load-bearing part of combustion chambers and high-temperature turbine blade of aeroengines. To reduce corrosion of the ceramic matrix composite (CMC) induced by water vapour in aeroengines [4], it is necessary to prepare an environmental barrier coating (EBC) system on its surface [5]. At present, rare-earth disilicates are the most promising EBC material. However, rare-earth disilicates are selectively corroded by high-temperature water vapour, which is one of the main causes of failure of EBC systems [6].

To characterise the corrosion behaviour of rare-earth disilicates under high-temperature water vapour, researchers usually use simple experimental devices, such as a thermal-steam cycling furnace [6,7], to simulate the gas environment of an engine gas. The rate of mass loss of various rare-earth monosilicates and disilicates can be obtained experimentally. However, the internal gas environment of an actual aeroengine in operation is extremely harsh, so it is difficult to completely simulate an aeroengine using experimental devices [7]. Design and construction of these experimental devices is hindered by high gas velocity, temperature, and pressure. Building an experimental platform with a gas environment similar to that of an aeroengine will require an enormous time and financial commitment [8]. In addition, the high-temperature atmosphere during the experiments often contains a certain amount of impurity gas, such as aluminium hydroxide generated from furnace with aluminium-oxide-tube, that interferes with the experimental results, altering the findings from the experiments. Therefore, the accuracy of the data obtained from experiments [9,10,11,12,13] is low and the conclusions drawn from them are highly uncertain. Moreover, owing to the complexity of the experimental device and its inherent limitations, the experimental time is usually limited to approximately 100 h [14,15], which is far less than the actual service time. Thus, it is impossible to explore the corrosion principle of rare-earth disilicates over a long period of time directly by experiments. Some theoretical models are used to explain and predict the corrosion behaviour of rare-earth disilicates under high-temperature water vapour, but at present, rare-earth disilicates can only be characterised qualitatively [16].

The purpose of this study is to develop a theoretical model to quantitatively describe the corrosion behaviour of rare-earth disilicates in high-temperature water vapour, and to predict the change of coating thickness or weight loss. The dependent factors, which include gas velocity, temperature, total pressure, and water pressure, are discussed and analysed. The model proposed in this study may provide better guidance for the design of EBC systems.

## 2. Conceptual Framework

The morphology, phase composition, and distribution of rare-earth disilicates corroded by water vapour have been reported by many researchers [6,10,11,12,13,14,15,16]. The model developed in this study is based on volume reduction-oxidation processes [17]. The process is very similar to the oxidation of traditional volume-shrinkage, such as the oxidation of zirconium boride (ZrB_2_) at high temperature. In this study, ytterbium disilicate is used as an example.

The corrosion process of ytterbium disilicate by water vapour at high temperature can be mainly described by the following chemical reaction:

Yb_2_Si_2_O_7_(s) + 2H_2_O(g) → Yb_2_SiO_5_(s) + Si(OH)_4_(g) (1)

where s represents solid state and g represents gas state. The surface of the ytterbium disilicate layer tends to corrode after long service in a high-temperature and high-velocity gas environment. The top of the residual coating has a loose porous structure. Water vapour causes loss of SiO_2_ in the disilicate crystal structure, and this produces volatile gaseous Si(OH)_4_, which can be removed by high-velocity gas. The severity of corrosion increases with the extension of service time.

**Figure 1:**
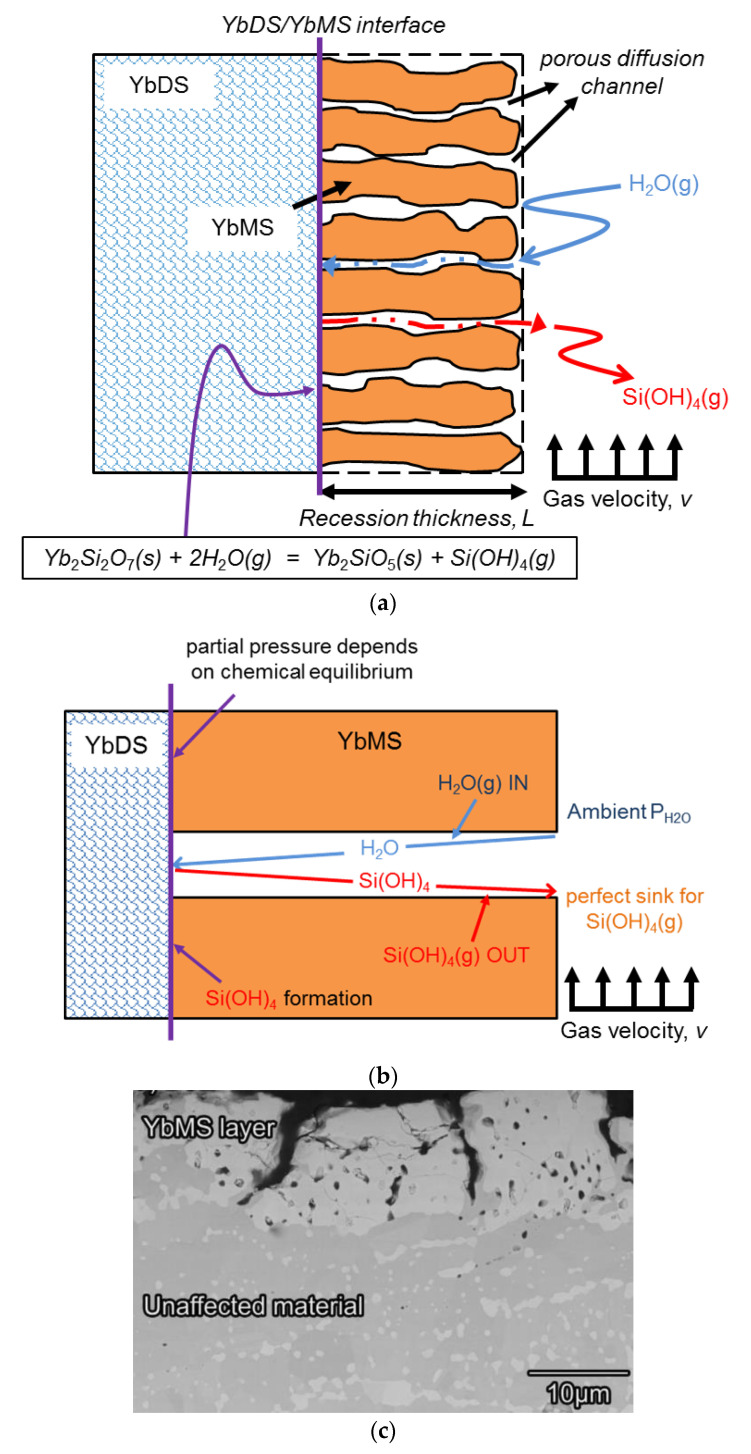
(**a**) Schematic of the corrosion process of ytterbium disilicate in an engine gas environment, (**b**) simplified model of the corrosion process, (**c**) the micrograph showing the corrosion process of environmental barrier coatings (EBCs) [6].

The schematic and a simplified model of the corrosion process of ytterbium disilicate are shown in Figure 1a,b, respectively. The orange region represents the ytterbium monosilicate (YbMS) left behind after corrosion by water vapour corrosion, while the blue region represents the intact ytterbium disilicate (YbDS). The velocity of the external gas that constantly scours the outer surface of the corroded area is constant. The water vapour in the external gas reaches the interface where the corrosion reaction takes place through the porous channel, and gaseous Si(OH)_4_ is generated and expelled. The purpose of the model in Figure 1b is to predict the thickness of the corrosion zone and the dynamic evolution of the weight of the material at different temperature and partial pressure of water vapour.

Some assumptions were made in this model. Since the porous channels provide a shortcut for the contact between the external environment and the interior of the coating, we suppose that diffusion of gas does not occur in the corroded area and occurs only in the pore channel. Ignoring the curvature, we assume that the porous channel is straight, and it has a continuous cylindrical shape. However, the size and diameter of the cylindrical channels are not consistent and uniform. The gases in the external gas environment consist of water vapour, oxygen, and fuel gases. Except for water vapour, the other gases do not chemically or physically interact with the rare-earth disilicates at the top of the coating. Therefore, we only considered the water vapour component in the external gas, and we did not discuss the influence of other components on the EBC system. It should be noted that the chemical reaction occurs during the corrosion process only at the interface of the corroded area. That is, the interface area was completely corroded by water vapour, which is only composed of monosilicates, while the uncorroded area still contains the original disilicates.

## 3. Establishment of the Theoretical Model

Under high-temperature gas environment, the reaction interface is filled with gaseous Si(OH)_4_, and porous ytterbium monosilicate is the main solid component of the products of corrosion. At the reaction interface, the concentration of the gaseous Si(OH)_4_ is higher and that of the water vapour. In the external gas, the gaseous Si(OH)_4_ concentration is lower, and the water vapour concentration is higher.

The difference in concentration is the driving force for the gas balance, and the mass transfer occurs between the two gases in the porous path of the top corroded area. Owing to the protection of the top corroded area, the high-velocity external gas is completely obstructed outside the coating system. The gas remains relatively static in the pores of the corroded area, with overall zero velocity. The mass transfer process that occurs in the corroded zone takes the form of molecular diffusion. The model in this study is based on quasi-static assumptions. According to Fick’s first law of diffusion [10]:

(2) $$J=-D\frac{\partial C}{\partial x}$$

where *J* represents the diffusion flux, the minus sign indicates an inverse concentration gradient diffusion, and *D* represents the molecular diffusion coefficient. *C* represents the concentration at a certain location, and its partial derivative represents the concentration gradient. All of the parameters used here are listed in Table 1.

Thus, the diffusion fluxes of water vapour and gaseous Si(OH)_4_ in the porous channel are given by:

(3) $$\begin{array}{l} J_{H_{2}O}=-fD_{H_{2}O}\frac{\partial C_{H_{2}O}}{\partial x}=fD_{H_{2}O}\frac{C_{H_{2}O}^{Q}-C_{H_{2}O}^{S}}{H}\\ J_{Si{\left(OH\right)}_{4}}=-fD_{Si{\left(OH\right)}_{4}}\frac{\partial C_{Si{\left(OH\right)}_{4}}}{\partial x}=fD_{Si{\left(OH\right)}_{4}}\frac{C_{Si{\left(OH\right)}_{4}}^{Q}-C_{Si{\left(OH\right)}_{4}}^{S}}{H} \end{array}$$

where *f* is the porosity of the corroded area (by surface), the subscript represents the corresponding gas type, while the superscript “*Q*” and “*S*” represent the chemical reaction interface and the surface of the corroded area respectively, and H is the thickness of the corroded area. The diffusion fluxes of water vapour and gaseous Si(OH)_4_ are negative and positive, respectively. Due to the quasi-static assumption, water vapour and gaseous Si(OH)_4_ do not accumulate at the reaction interface; thus, the diffusion flux of water vapour and gaseous Si(OH)_4_ will satisfy the following equation:

(4) $$J_{H_{2}O}+2\cdot J_{Si{\left(OH\right)}_{4}}=0$$

The gas pressure is used to control the concentration of gas in the experiment where water vapour is used to corrode EBC. In the gas phase, the gas component is generally characterised by the gas partial pressure. From the ideal gas law, Equation (4) can be rewritten in the form of gas pressure:

(5) $$\begin{array}{l} P=R\cdot T\cdot C\\ {\left|J_{H_{2}O}\right|}_{\mathrm{Inside}}=fD_{H_{2}O}\frac{C_{H_{2}O}^{R}-C_{H_{2}O}^{S}}{H}=fD_{H_{2}O}\frac{P_{H_{2}O}^{S}-P_{H_{2}O}^{Q}}{R\cdot T\cdot H}\\ {\left|J_{{\mathrm{Si}(\mathrm{OH})}_{4}}\right|}_{\mathrm{Inside}}=fD_{{\mathrm{Si}(\mathrm{OH})}_{4}}\frac{C_{{\mathrm{Si}(\mathrm{OH})}_{4}}^{R}-C_{{\mathrm{Si}(\mathrm{OH})}_{4}}^{S}}{H}=fD_{{\mathrm{Si}(\mathrm{OH})}_{4}}\frac{P_{{\mathrm{Si}(\mathrm{OH})}_{4}}^{Q}-P_{{\mathrm{Si}(\mathrm{OH})}_{4}}^{S}}{R\cdot T\cdot H} \end{array}$$

where “Inside” refers to the interior of the corroded area, *P* is the pressure of the gas, *R* is the molar gas constant (8.31 J/(mol·K)), and *T* is the absolute temperature.

Equation (5) is only used to describe the molecular diffusion process in the internal gas channel. Next, we need to consider the influence of the external gas environment on the diffusion process. The mass transfer in the fluid caused by macroscopic motion is significant. Mass transfer occurs by convection in the gaseous region of the external environment. Convective mass transfer depends not only on molecular diffusion but also on the macro motion of the fluid. The mass transfer rate is generally greater than that of molecular diffusion. Then, the diffusion fluxes of water vapour and gaseous Si(OH)_4_ in the external flow gas, which are determined by the boundary layer thickness, the gaseous diffusivity, and the gaseous diffusivity, are given by:

(6) $$\begin{array}{l} {\left|J_{H_{2}O}\right|}_{\mathrm{Outside}}=D_{H_{2}O}\frac{C_{H_{2}O}^{A}-C_{H_{2}O}^{S}}{\delta_{H_{2}O}}=D_{H_{2}O}\frac{P_{H_{2}O}^{A}-P_{H_{2}O}^{S}}{RT\delta_{H_{2}O}}\\ {\left|J_{{\mathrm{Si}(\mathrm{OH})}_{4}}\right|}_{\mathrm{Outside}}=D_{{\mathrm{Si}(\mathrm{OH})}_{4}}\frac{C_{{\mathrm{Si}(\mathrm{OH})}_{4}}^{S}-C_{{\mathrm{Si}(\mathrm{OH})}_{4}}^{A}}{\delta_{{}_{{\mathrm{Si}(\mathrm{OH})}_{4}}}}=D_{{\mathrm{Si}(\mathrm{OH})}_{4}}\frac{P_{{\mathrm{Si}(\mathrm{OH})}_{4}}^{S}-P_{{\mathrm{Si}(\mathrm{OH})}_{4}}^{A}}{RT\delta_{{}_{{\mathrm{Si}(\mathrm{OH})}_{4}}}} \end{array}$$

where “Outside” refers to the external gas environment and superscript “*A*” represents the turbulent core area in the ambient. The boundary layer thicknesses are given by Reference [18]:

(7) δH2O=1.5L(Re)−1/2(Sc)−1/3=1.5L(vρLη)−1/2(ηDH2Oρ)−1/3δSi(OH)4=1.5L(vρLη)−1/2(ηDSi(OH)4ρ)−1/3

where *ρ*, *V*, and *η* refer to the density, velocity, and viscosity of the fluid respectively, and *L* is the characteristic length of EBCs exposed to external conditions. Under the quasi-static assumption, the gas diffusion flux in the external gas should be similar to the gas diffusion flux in the corroded area; therefore, Equations (5) and (6) can be written as follows:

(8) $$\begin{array}{ll} {\left|J_{H_{2}O}\right|}_{\mathrm{Inside}} & ={\left|J_{H_{2}O}\right|}_{\mathrm{Outside}}\\ & =fD_{H_{2}O}\frac{P_{H_{2}O}^{S}-P_{H_{2}O}^{R}}{RTH}=D_{H_{2}O}\frac{P_{H_{2}O}^{A}-P_{H_{2}O}^{S}}{RT\delta_{H_{2}O}}\\ {\left|J_{{\mathrm{Si}(\mathrm{OH})}_{4}}\right|}_{\mathrm{Inside}} & ={\left|J_{{\mathrm{Si}(\mathrm{OH})}_{4}}\right|}_{\mathrm{Outside}}\\ & =fD_{{\mathrm{Si}(\mathrm{OH})}_{4}}\frac{P_{{\mathrm{Si}(\mathrm{OH})}_{4}}^{R}-P_{{\mathrm{Si}(\mathrm{OH})}_{4}}^{S}}{RTH}=D_{{\mathrm{Si}(\mathrm{OH})}_{4}}\frac{P_{{\mathrm{Si}(\mathrm{OH})}_{4}}^{S}-P_{{\mathrm{Si}(\mathrm{OH})}_{4}}^{A}}{RT\delta_{{\mathrm{Si}(\mathrm{OH})}_{4}}} \end{array}$$

Then, the equation of the rate at which the thickness of the corroded area changes and the loss of mass per unit area can be obtained by accounting for the flux balance using Equation (4), which can be written as follows:

(9) $$\frac{dH}{dt}=\left(\frac{1}{1-f}\right)\frac{1}{\rho_{\mathrm{YbMS}}}\frac{dW_{\mathrm{YbMS}}}{dt}=\left(\frac{J_{{\mathrm{Si}(\mathrm{OH})}_{4}}}{1-f}\right)\frac{M_{\mathrm{YbMS}}}{\rho_{\mathrm{YbMS}}}=\left(\frac{1}{1-f}\right)\left(\frac{1}{2}\left|J_{H_{2}O}\right|\right)\frac{M_{\mathrm{YbMS}}}{\rho_{\mathrm{YbMS}}}$$

(10) $$\frac{dR}{dt}=\frac{dH}{dt}(1-f)\frac{M_{\mathrm{YbDS}}/\rho_{\mathrm{YbDS}}}{M_{\mathrm{YbMS}}/\rho_{\mathrm{YbMS}}}$$

(11) $$\frac{\Delta W}{S}=H(1-f)\rho_{\mathrm{YbMS}}-R\rho_{\mathrm{YbDS}}$$

Integration of Equation (9) gives a parabolic equation that represents the growth of the corroded area:

(12) $$\begin{array}{l} H=\sqrt{2At+B^{2}}-B\\ A=\frac{f}{2\left(1-f\right)}\cdot \frac{M_{\mathrm{YbMS}}}{\rho_{\mathrm{YbMS}}}\cdot \frac{D_{H_{2}O}\left(P_{H_{2}O}^{A}-P_{H_{2}O}^{R}\right)}{RT}\\ B=f\delta_{H_{2}O} \end{array}$$

The chemical reaction equilibrium constant of the corrosion process is expressed using the partial pressure of gaseous H_2_O and Si(OH)_4_:

(13) $$K_{{\mathrm{SiO}}_{2}}\cdot a_{{\mathrm{SiO}}_{2}}=P_{{\mathrm{Si}(\mathrm{OH})}_{4}}^{R}/{\left(P_{H_{2}O}^{R}\right)}^{2}$$

where $K_{SiO_{2}}$ is the chemical equilibrium constant of the reaction between SiO_2_ and water vapour, and $a_{SiO_{2}}$ is the activity of silica, which represents the difficulty of the chemical reaction between the rare-earth silicate and water vapour. $K_{SiO_{2}}$ and $a_{SiO_{2}}$ at the reaction interface of the corroded area is given by References [19,20]:

(14) $$\mathrm{lg}a_{{\mathrm{SiO}}_{2}}=-(2216\pm 161)T^{-1}+0.81\pm 0.09$$

(15) $$\mathrm{lg}K_{{\mathrm{SiO}}_{2}}=-2851.2T^{-1}-3.5249$$

The diffusion coefficient of gaseous H_2_O and Si(OH)_4_ that diffused through the porous region is to be determined. The diffusion coefficient in a multi-component gaseous system can be approximated by Reference [21]:

(16) $$D_{1,(2,\ldots ,i)}=\frac{1}{\sum_{i}^{i\neq 1}x_{i}/D_{1-i}};x_{i}=\frac{n_{i}}{\sum_{j}^{j\neq 1}n_{j}}$$

where *n* is the mole fraction, the subscript ‘‘1, (2,..., i)’’ is the diffusivity of species 1 in a multi-component mixture, and the subscript ‘‘1 − *i*’’ is the diffusivity of species 1 in a binary mixture of species 1 and *i*. According to the Chapman–Enskog equation, the diffusivity D_1–2_ is given by Reference [22]:

(17) $$D_{1–2}=\frac{0.0018583T^{3/2}\sqrt{\left(1/M_{1}\right)+\left(1/M_{2}\right)}}{r_{12}^{2}\Omega_{D}P}$$

(18) $$\Omega_{D}=\frac{1.06036}{{\left(T^{*}\right)}^{0.15610}}+\frac{0.193}{\exp(0.47635T^{*})}+\frac{1.03587}{\exp(1.52996T^{*})}+\frac{1.76474}{\exp(3.89411T^{*})}$$

where *M* is the molecular weight, Ω is the gas molecule collision integral, and *r*_12_ is the average collision diameter between gas molecules. The parameters required for the above expression can be obtained from the study by Svehla [23]. Since these parameters are not available for gaseous Si(OH)_4_, values for SiF_4_ were used as an approximation. Krikorian [24] noted that hydroxides act as pseudo halides, and this suggests that the aforementioned approximation is reasonable.

## 4. Results

In this section, the corrosion of ytterbium disilicate by water vapour in a high-temperature water and oxygen environment was analysed. Since the model contains several variables, the control variable method was used to determine and discuss the influence of a single parameter on the corrosion process. The values of other fixed gas-related parameters are as follows [25]: the gas viscosity was 5.44 × 10^−4^, the gas density was 1.4 × 10^−3^, and the surface characteristic length of the coating sample was 2.5 cm.

### 4.1. Influence of Gas Temperature on the Corrosion Process

**Figure 2:**
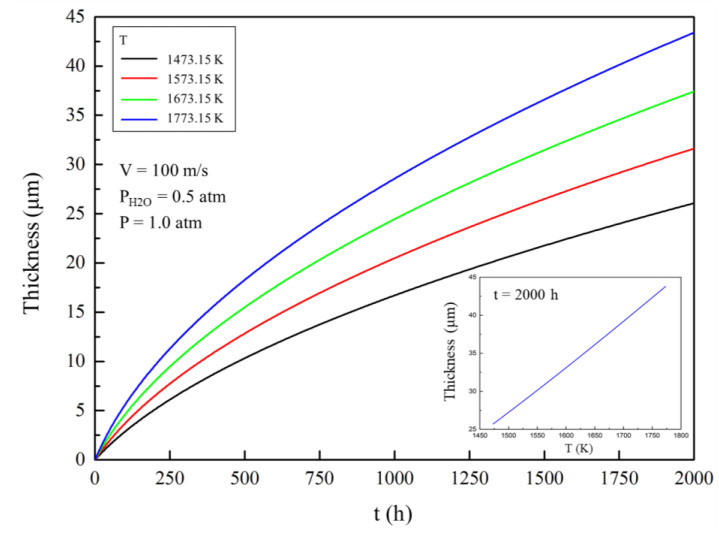
Thickness evolution of the ytterbium disilicate (YbDS) corroded area with time at different external gas temperature (while the inserted graph shows the relationship between the thickness of the ytterbium disilicate (YbDS) corroded area and the temperature of external gas after 2000 h of corrosion).

In this section of the study, the velocity of external gas was 100 m/s, the partial pressure of water vapour in the gas environment was 0.5 atmosphere, the total gas pressure was 1.0 atmosphere, and the porosity in the corroded area was 0.26 [6]. Figure 2 shows the change in the thickness of the corroded area with time in different external gas temperatures. The thickness of the corroded area in the EBC gradually increases with the increase of the corrosion time in a high-temperature gas environment, which means that the mass loss per unit area gradually increases. The relationship between the corrosion time and the thickness of the corroded area is not strictly linear, but a similar parabola. Thickness growth rate and mass loss rate per unit area decrease with time.

When the gas temperature increases, the corrosion rate of ytterbium disilicate increases. The thickness of the corroded area after 2000 h is approximately 20 μm and over 40 μm, at a temperature of 1473.15 K (1200 °C) and 1773.15 K (1500 °C), respectively.

Considering the gas velocity, water pressure, and total pressure, the corrosion process was very sensitive to temperature change. This is mainly caused by the following two reasons: (i) Increase of the external temperature accelerates the chemical reaction, which raises the partial pressure of Si(OH)_4_ at the corrosion reaction interface, thus improving the concentration, which is the driving force of molecular diffusion, and (ii) increase of the external temperature accelerates the thermal motion of gas molecules, which increases the diffusion coefficient. The increase in temperature also increases the probability of intermolecular collision of gas molecules and inhibits gas diffusion to some extent. The overall effect of the accelerated Brownian motion also favours the diffusion of gas. Owing to the aforementioned reasons, the gas diffusion flux increases, which ultimately accelerates the evaporation rate of the coating.

Under the same external gas parameters, the relationship between the thickness of the corroded area and the gas temperature after 2000 h is also shown in Figure 2. In the range of 1473.15 (1200 °C) to 1773.15 K (1500 °C), the curve is approximately a straight line, indicating that the thickness of the corroded area is approximately proportional to the gas temperature.

### 4.2. Influence of External Gas Velocity on the Corrosion Process

**Figure 3:**
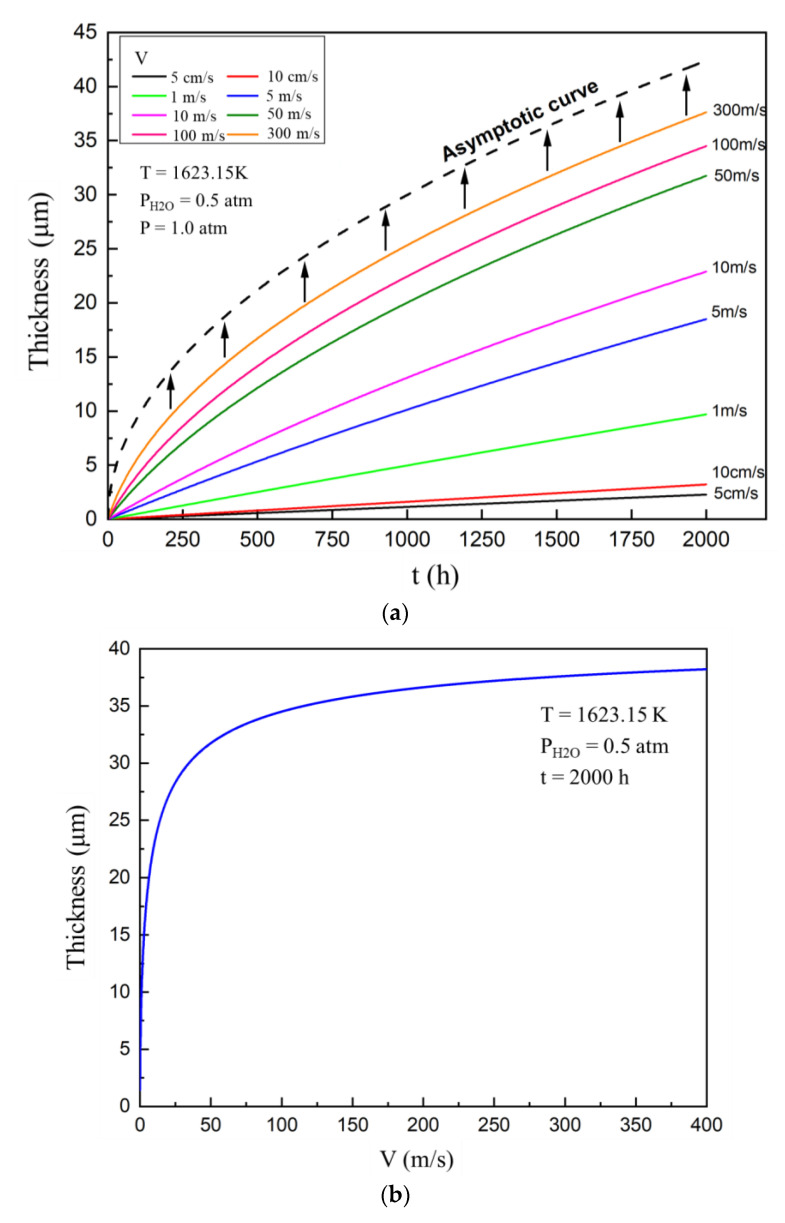
(**a**) Evolution of the thickness of ytterbium disilicate (YbDS) corroded area with time at different gas velocity. (**b**) The relationship between the thickness of ytterbium disilicate (YbDS) corroded area and the gas velocity after 2000 h of corrosion.

In this section of the study, the temperature of the external gas was 1623.15 K (1350 °C), the total gas pressure was 1.0 atmosphere, the water pressure was 0.5 atmosphere, and the porosity of the corroded area was 0.26. Figure 3a shows that the thickness of the corroded area changed with time under different external gas velocities. The results obtained from calculation show that the corroded area gradually thickens at both low- and high-velocity; however, different gas flow velocities significantly influence the corrosion process.

The gas flow velocity used in most of the simplified experimental equipment, such as the tubular high-temperature muffle furnace, was approximately 10 cm/s or less [25]. At low gas flow velocity, the thickness evolution curve of the corroded area is an approximately straight line, and the thickening rate of the corroded area is relatively low. However, the thickening rate of the corroded area increased with a gradual increase of the external gas velocity. When the gas velocity was increased above 50 m/s, the corrosion curve began to bend significantly, forming a parabolic shape. As the flow velocity of the external gas was further increased to hundreds of meters per second, which corresponded to the real working condition of EBCs in an aeroengine, the corrosion process further accelerated until an asymptotic parabolic curve was formed. The increase of the external flow velocity obviously promotes the corrosion of ytterbium disilicate, but there is a limit to which this gas flow velocity promotes this corrosion process effect. At this limit, the corrosion curve does not exceed the range of the asymptote.

Under the same external gas parameters, the relationship between the thickness of the corroded area after 2000 h and the external gas velocity is shown in Figure 3b. There is a velocity boundary value in the velocity range of the external gas. When the gas velocity was less than 50 m/s, the corrosion process was very sensitive to the external velocity. Even a relatively small increase or decrease in velocity resulted in a significant change in the thickness of the corroded area. However, when the flow velocity was greater than 50 m/s, the effect of the corrosion process on the coating was significantly lowered. Finally, at an ultra-high gas flow velocity, the effect of the external gas flow velocity was insignificant.

When the velocity of external gas was low, the Reynolds number was low, and the flow was stable. The water vapour and gaseous Si(OH)_4_ on the outer surface have thicker boundary layers. The increase of the mass transfer distance lowers the mass transfer effect of the external gas and it also lowers the gas flux, resulting in a slower corrosion process at low flow velocity. The inverse happened when the external gas velocity was high. The boundary layer thickness approached zero as the external gas velocity increased, but the gas channel in the corroded area of the coating was still present. The corrosion phenomenon was eventually completely controlled by the gas channel in the corroded area, which was not affected by the external flow velocity. This limiting case corresponds to the asymptotic curve in Figure 3a. The expression of the curve is as follows:

(19) $$H=\sqrt{2At}\;A=\frac{f}{2\left(1-f\right)}\cdot \frac{M_{\mathrm{YbMS}}}{\rho_{\mathrm{YbMS}}}\cdot \frac{D_{H_{2}O}\left(P_{H_{2}O}^{A}-P_{H_{2}O}^{R}\right)}{RT}$$

### 4.3. Influence of Water Pressure on the Corrosion Process

**Figure 4:**
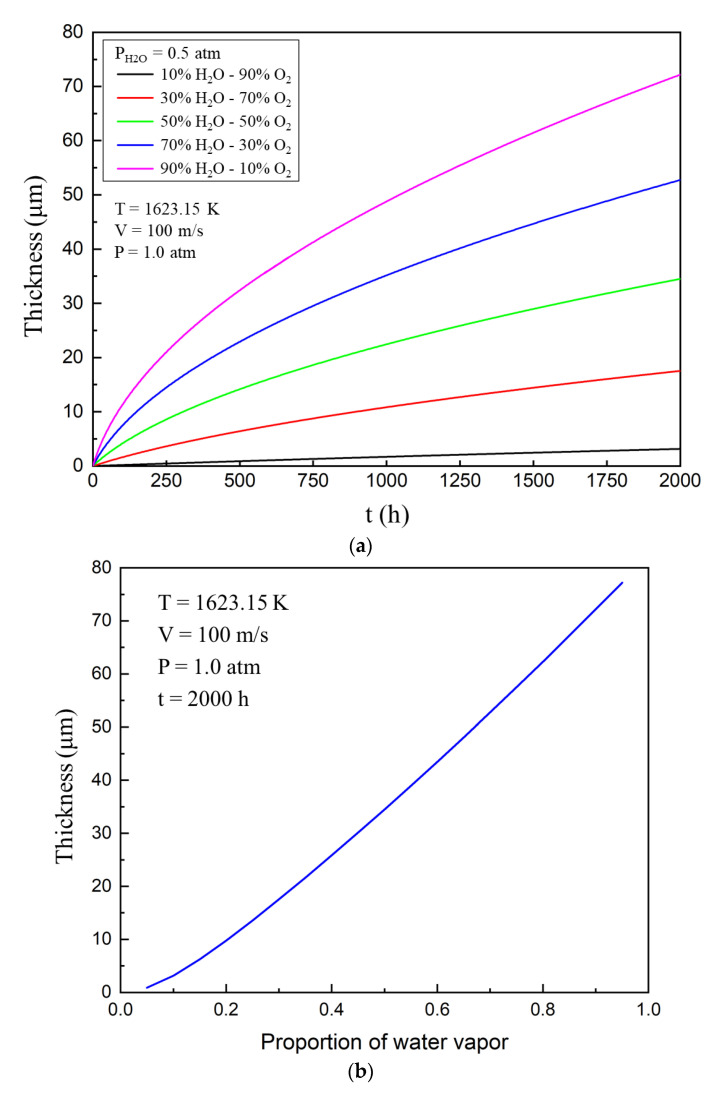
(**a**) Evolution of the thickness of ytterbium disilicate (YbDS) corroded area with time at different water vapour pressures. (**b**) The relationship between the thickness of ytterbium disilicate (YbDS) corroded area and the water pressure after 2000 h of corrosion.

In this section of the study, the external gas temperature was 1623.15 K (1350 °C), gas velocity was 100 m/s, total gas pressure was 1.0 atmosphere, and porosity was 0.26. Figure 4a shows that the thickness of the corroded area changes with time at different water pressure. When the ratio of water to oxygen is low, the corrosion rate of ytterbium disilicate is slow and the corrosion curve is close to a straight line. When the ratio of water to oxygen is gradually increased, the corrosion rate increases gradually. Meanwhile, the corrosion curve gradually deviated from the straight line and became parabolic. It can be seen that the corrosion process is also very sensitive to the water pressure of the external gas.

The concentration of reactants during corrosion increases with the increase in water pressure, and this accelerates the rate of the chemical reaction. Gaseous Si(OH)_4_ is a product of the chemical reaction, and its concentration or partial pressure at the corrosion interface also rises, thus increasing the concentration driving force of gas diffusion, and accelerating the corrosion rate of the EBC system. It can be seen from Figure 4b that the thickness of the corroded area increases significantly with an increase in water pressure. The relation between corrosion depth and water pressure is almost proportional.

### 4.4. Influence of Total Pressure on the Corrosion Process

**Figure 5:**
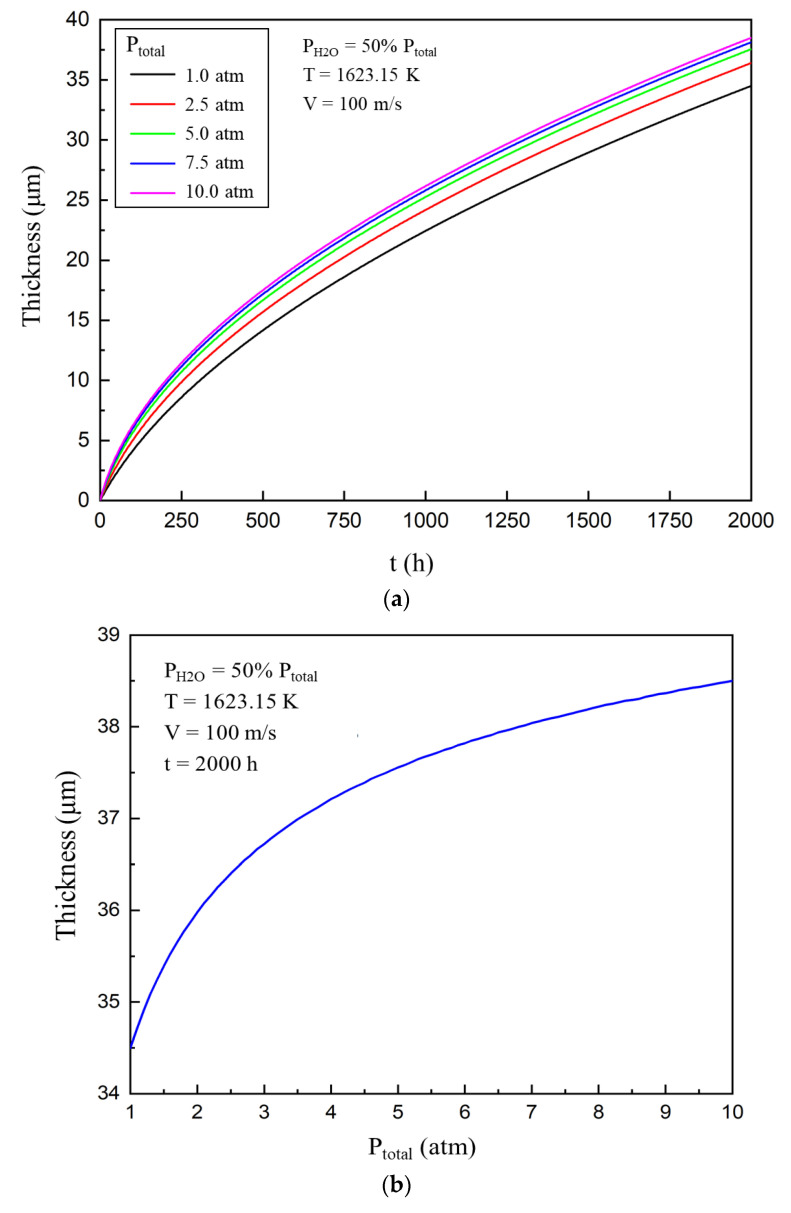
(**a**) Evolution of the thickness of ytterbium disilicate (YbDS) corroded area with time at different total pressure. (**b**) Relationship between the thickness of ytterbium disilicate (YbDS) corroded area and the total pressure after 2000 h of corrosion.

In this section of the study, the external gas temperature was 1623.15 K (1350 °C), gas velocity was 100 m/s, water pressure was set at half of the total pressure, and porosity was 0.26. Figure 5a shows the change in the thickness of the corroded area with time at different total pressures. The corrosion process can be accelerated by increasing the total pressure of the external gas. It can be seen from Figure 5b that a lower total pressure of the external gas promotes the corrosion process, while an increase in pressure weakly affects the process. Two major factors jointly affect the corrosion process. First, increasing the total gas pressure accelerates the chemical reaction rate. Second, an increase in the total gas pressure increases the gas molecular density, which hinders the diffusion of the gas and reduces the molecular diffusion coefficient of the gas, thus reducing the gas diffusion flux. There are mutual restrictions and competition between the molecular diffusion coefficient and the gas diffusion flux. For ytterbium disilicate, the acceleration effect of the former is stronger than the mitigation effect of the latter, so the increase of total pressure will slightly promote its corrosion.

### 4.5. Influence of Different Parameters on the Corrosion Kinetics Curve

**Figure 6:**
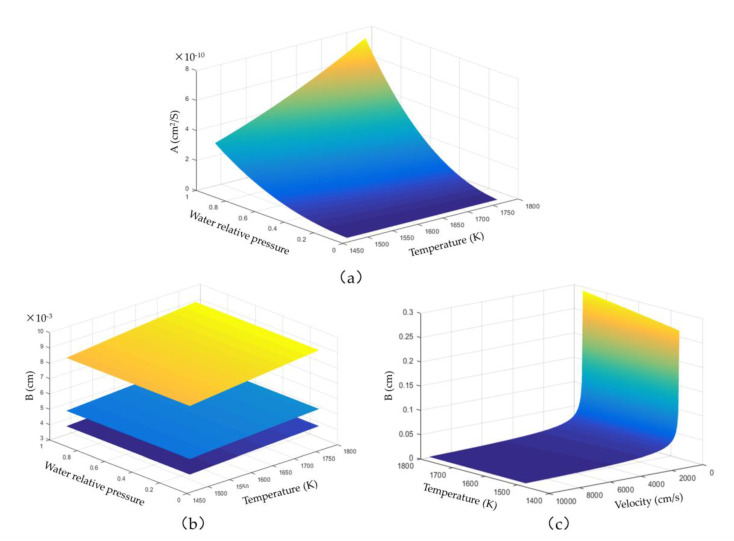
Relationship of each parameter with variables A and B: (**a**) Influence of temperature and water pressure on variable A, (**b**) influence of temperature, water pressure, and total pressure on variable B, (**c**) influence of temperature and flow velocity on variable B.

The corrosion of rare-earth disilicate coatings under high-temperature water vapour is affected by several factors, such as gas temperature, gas velocity, and gas water pressure. The shape of the corrosion curve is different under different conditions. From Equation (12), it was found that the corrosion curve forms a translational parabola. The shape of the corrosion curve depends entirely on the variables “A” and “B”. Because variable “A” corresponds to the diffusion process in the corroded area, the other three external factors, except for the external velocity, have significant influence on this variable. It can be seen in Figure 6 that the effect of temperature on variable “A” depends on the water pressure of the gas. When the water pressure is low, the effect of temperature change is very weak, but when the water pressure is high, the effect of temperature change is obviously enhanced. Variable “A” is more sensitive to changes in the partial pressure of water vapour than in temperature.

The variable “B” represents the diffusion process in the external gas boundary layer. The gas velocity and temperature affect the shape of the curve. It can be seen from Figure 6c that the effect of temperature is insignificant at high flow velocity. The partial pressure of water vapour has little effect on variable “B”; however, an increase of the total pressure of the gas increases its partial pressure. In general, the shape of the corrosion curve mainly depends on the combination of “A” and “B”. The corrosion curve is a straight line only when the temperature, water pressure, and flow velocity of gas are low, and the total pressure of the gas is high. Otherwise, the corrosion curve has a parabolic shape.

### 4.6. Comparative Analysis of the Model and Experimental Results

Researchers in the National Aeronautics and Space Administration (NASA) investigated the corrosion volatilisation of ytterbium disilicide under high-temperature water vapour environment using thermogravimetric analysis [25]. Firstly, the ytterbium disilicate powder with a particle size of 1–5 μm was hot-pressed in vacuum at 1500 °C/27.58 MPa, and block samples with a size of 2.5 × 1.25 × 0.15 cm^3^ were obtained. To simulate the combustion environment in the engine, the samples were placed in a tube muffle furnace at 1500 °C with a total gas pressure of 1.0 atmosphere. Then, a mixture of 50% H_2_O and 50% O_2_ was injected into the high-temperature alumina furnace tube at a flow velocity of 4.4 cm/s. The results of the experiment conducted by NASA showed the mass loss of the samples.

**Figure 7:**
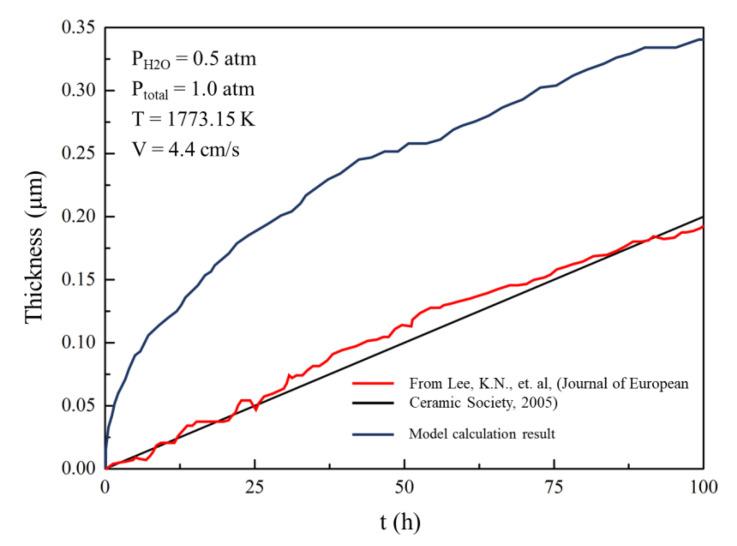
Comparison of the model data and the experimental data. The experimental data were obtained from a study conducted by NASA [25].

For convenience, Equations (10) and (11) were used to convert the reduction in mass to the thickening of the corroded area, as shown in Figure 7. It should be noted that the sample is a long flake, and the upper and lower surfaces can be corroded by water vapour. The reduction in mass of the experimentally measured sample is approximately equal to the sum of the thickness of the upper and lower surfaces. The corrosion model used in this study only considers one side of the surface, thus the experimental data value is half of the original value.

The model results are in good agreement with the experimental results, and the curves are approximately straight lines. This is because at low flow velocity, the boundary layer is thick. In addition, under the same experimental conditions, the experimental results of another group differ significantly from those obtained using this model. The thickness growth curve is parabolic, and the effect of corrosion was more intense. This indicates that turbulence accounts for a large part of the gas, which makes the boundary layer thinner. This phenomenon may be related to the surface roughness and the placement of the sample. Rougher sample surfaces make the gas flow unstable at low flow velocity. In addition, if the short side of the long-flake sample is parallel to the direction of gas flow, the characteristic length will be shorter, which makes it difficult to form a thicker boundary layer.

## 5. Conclusions

A theoretical model for predicting the corrosion behaviour of rare-earth disilicates in high-temperature water vapour was established based on the gas diffusion theory. The influence of external gas parameters on the corrosion behaviour was discussed. The molecular diffusion inside the corroded area and the mass transfer process near the surface of the corroded area were considered in the model used for this study, which is in reasonable agreement with the experimental data obtained from other literature. The main conclusions drawn from this study are as follows:

(1) The corrosion depth evolution curve of the rare-earth disilicate EBC system under high-temperature water vapour is parabolic. The shape of the thickening curve depends on the combination of several parameters. The corrosion curve is linear only when the temperature, water pressure, and flow velocity of gas are low, and the total pressure of gas is high. Otherwise, the curve is parabolic.
(2) The increase of external gas temperature, flow velocity, water vapour pressure, and total pressure can accelerate the corrosion rate.
(3) The degree of influence of the external gas parameters varies: the gas velocity and water pressure have the greatest influence on the corrosion process, followed by gas temperature, while the total gas pressure has the least influence in the corrosion process.

## Figures and Tables

**Table 1:** Summary of the used parameters and corresponding physical meanings.

Symbol	Property	Units
*J*	Diffusion flux	m^3^/s
*D*	Molecular diffusion coefficient	m^2^/s
*C*	Diffusion concentration at a certain location	m^2^/s
*f*	Porosity of the corroded area	%
*H*	Thickness of the corroded area	m
*P*	Pressure of the gas	Pa
*R*	Molar gas constant	J/(mol·K)
*T*	Absolute temperature	K
*ρ*	Density of the fluid	10^3^ Kg/m^3^
*V*	Velocity of the fluid	m/s
*η*	Viscosity of the fluid	mPa·s
*K_SiO_*_2_	Chemical equilibrium constant of the reaction between SiO_2_ and water vapour	—
*α* *_S_* *_i_* *_O_*_2_	Activity of silica	—
*M*	Molecular weight	g/mol
*Ω*	Gas molecule collision integral	—
*r*_12_	average collision diameter between gas molecules	Å (10^−10^ m)
